# Pyrolyzed “Black Mass” Feedstocks and Their Synthetic Proxies Relevant to Li-Ion Battery Recycling

**DOI:** 10.1021/acsomega.5c00995

**Published:** 2025-06-10

**Authors:** Alexander J. Bologna, Rebecca C. Vincent, Anna Kallistova, Justin A. Mayer, Matthew A. Wright, Clarina R. Dela Cruz, Rui Zhang, Fabian Seeler, Kerstin Schierle-Arndt, Ram Seshadri

**Affiliations:** † Department of Chemistry & Biochemistry, 8786 University of California Santa Barbara, California 93106, United States; ‡ Materials Research Laboratory, University of California, Santa Barbara, California 93106, United States; § Materials Department, University of California, Santa Barbara, California 93106, United States; ∥ Neutron Scattering Division, Oak Ridge National Laboratory, 1 Bethel Valley Rd., Oak Ridge, Tennessee 37831, United States; ⊥ California Research Alliance (CARA), BASF Corporation, Berkeley, California 94720, United States; # 5184 BASF SE, Ludwigshafen 67056, Germany

## Abstract

Lithium-ion battery (LIB) recycling aims to recover valuable materials present within end-of-life electrochemical cells. Industrial recycling processes produce “black mass” from recycling feedstock from which desirable materials can be recollected. Spent cells first undergo mechanical shredding and sieving, and organic components are removed by thermal treatment (pyrolysis) before hydrometallurgical processing is employed to recover the constituent elements. Black mass may contain a range of reaction products, formed at high temperature during pyrolysis, due to the compositionally complex and inhomogeneous nature of recycling feedstock. These products, however, may have different elemental compositions, ratios, and structures, making efficient hydrometallurgical recovery difficult. Here, we present three distinct, industrially sourced black mass samples containing Li­(Ni_*x*_Mn_*y*_Co_*z*_)­O_2_ (*x* + *y* + *z* = 1) positive electrodes of varying composition. We employ a suite of structural and compositional characterization techniques, including synchrotron X-ray and neutron powder diffraction and element specific analysis (X-ray photoelectron spectroscopy, X-ray fluorescence spectroscopy, energy dispersive X-ray spectroscopy, inductively coupled plasma optical emission spectroscopy), to identify phases formed during commercial treatment of recycling feedstocks and how their relative quantities are affected by process order. Additionally, we also present results of studies on simpler model systems to better identify minor phases present within the complex recycling feedstocks and to direct the efficient recovery of valuable components.

## Graphical abstract

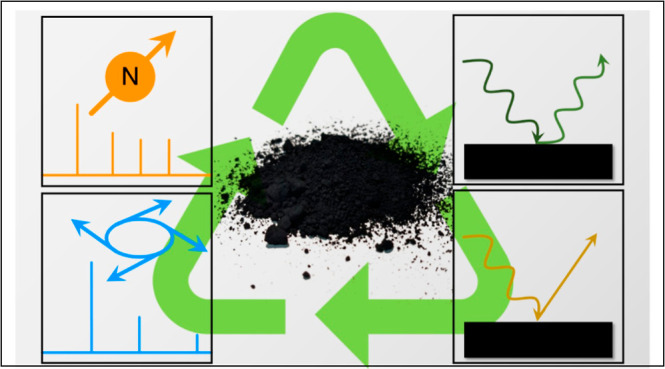

## Introduction

Lithium-ion batteries (LIBs) facilitate high-performance portable electronic devices and electric vehicles, synonymous with modern life.^1,2^ Despite their societal prevalence and commercial success, there are growing concerns regarding the multifarious social, ethical, and environmental issues surrounding LIB technology. Many of the components in LIBs are scarce, hazardous, or toxic.^3,4^ Resource scarcity is a significant obstacle to the wider employment of LIB technology, with concerns regarding the inhomogeneous geological availability, geopolitics, and market limitations of sources of Li, Co, and natural graphite.^5–7^ Furthermore, improper disposal of LIBs in landfills poses serious environmental and safety risks associated with the leaching of cobalt and fluorine containing compounds,^8^ fire hazards,^9^ and toxic gas release.^5,10,11^ While there is a clear need for the widely employed and effective recycling of spent LIBs, there remains a lack of compositional understanding of the complex blend of products created during LIB recycling processes. Development of such an understanding is necessary to improve recycling efficacy and continue to meet growing global energy requirements sustainably and responsibly.

A variety of LIB recycling strategies have been developed to address these challenges, with the three primary approaches being pyrometallurgical, hydrometallurgical, and direct recycling.^12,13^ Pyrometallurgical recycling involves high-temperature smelting processes that reduce transition metals (Co, Ni, Cu) into alloys while burning off organics. Although effective for metal recovery, pyrometallurgical approaches typically sacrifice graphite, lithium, and aluminum recovery and consume significant energy.^14^ Hydrometallurgical recycling relies on the chemical leaching of black mass using acids or bases, followed by selective precipitation or solvent extraction to recover individual elements. This approach allows for greater material specificity and lower temperatures but often generates complex wastewater and requires extensive pretreatment. Direct recycling aims to recover the functional structure of cathode materials without breaking them down into elemental constituents. While promising in its potential to preserve material value and reduce processing intensity, direct recycling is highly sensitive to feedstock variation and remains technologically immature at scale.^15^ These three approaches offer different balances among energy input, material recovery efficiency, and sensitivity to input variability, but hydrometallurgical approaches currently dominate the industrial LIB recycling landscape.

While there are a variety of flowsheets used in hydrometallurgical recycling, most routes require pretreatment of the LIB feedstock. The recycling plant will generally start by discharging (removing lithium from the graphitic anode) the LIBs for safety,^6^ followed by a combination of mechanical, thermal, and hydrometallurgical techniques^6,8,16,17^ employed to recover valuable components. Mechanical shredding exposes the various components for chemical reactions, while sieving is used to separate the larger pieces (current collector foils like Al and Cu as well as metallic casings) from the smaller particles comprising the active cathode materials (oxides of Li, Ni, Mn, and Co), graphitic anodes, and electrolyte/binder residues (containing C, F, and P). Collectively, this powered material is named “black mass” for its appearance. Thermal treatment of the black mass (ca. 100 °C to 300 °C) is employed to eliminate organic electrolyte solvents. Alternatively, pyrolysis (ca. 500 °C to 700 °C) is selected to thermally decompose binding polymers. Ideally, pyrolysis would never exceed the melting point of the Al metal (660 °C). These steps are necessary to ensure organic species are removed from black mass, the presence of which is deleterious to the subsequent hydrometallurgical steps and leads to wastewater that is expensive to remediate.^18,19^ Finally, hydrometallurgical techniques are employed to selectively precipitate desired metal salts following dissolution in acid.^20,21^ Figure 1 illustrates just some of the wide range of materials present in black mass after recycling processes. Lithium and transition metals have been recovered with high yields at the laboratory scale.^6,22–24^ However, proper sorting of waste streams and cell disassembly is not feasible beyond the laboratory setting.^25,26^ In practice, these studies present limited industrial relevance; waste feedstocks are less homogeneous on an industrial scale, with a wide range of cell configurations, component materials, and chemistries. While lab-scale experiments have provided some insight into the complex chemistries occurring during LIB recycling, it is necessary to develop an understanding of how industrial processing of bulk waste streams influences the constituent elemental and phase compositions of black mass.

**Figure 1:**
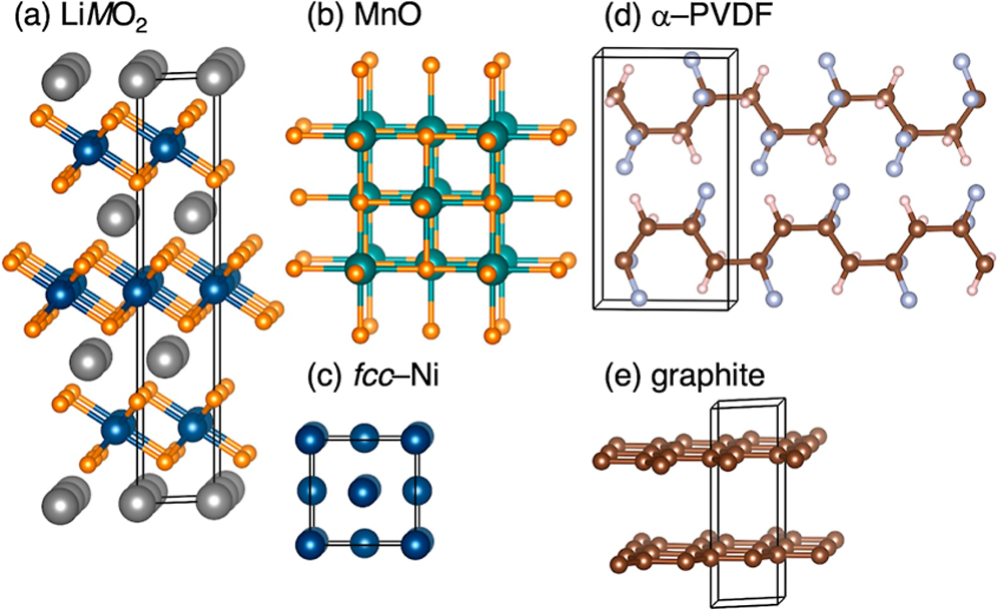
Crystal structures of common component phases within industrial black mass compositions. (a) 3R-LiMO_2_ (M = Mn, Co, Ni), (b) rock-salt MnO, (c) FCC-Ni (which forms upon heating), (d) α-PVDF, and (e) graphite.

Here, we present detailed structural and elemental analysis of three different industrial pyrolyzed black mass samples and characterize their constituent phase compositions. We used X-ray diffraction (XRD) and neutron diffraction to identify phases present and scanning electron microscopy (SEM) coupled with energy dispersive X-ray spectroscopy (EDS), X-ray fluorescence (XRF), X-ray photoelectron spectroscopy (XPS), and inductively coupled plasma–optical emission spectroscopy (ICP–OES) to identify their elemental compositions. To identify trace phases, we also present synthetic proxies, resulting from pyrolysis of pairs of battery components, as a means of better understanding what occurs in industrial-scale systems. By understanding the constituents in industrial samples—with insights from these lab-based models—we aim to inform the design of hydrometallurgical recovery of the valuable LIB components beyond the laboratory scale.

## Experimental Methods

### Materials

#### As-Received Black Mass

The subject of this study included three black mass samples from an industrial LIB recycling facility. Each sample was processed differently, as shown in Figure 2. Sample #1 was shredded, sieved, and vacuum-dried but not pyrolyzed. Sample #2 was mechanically processed in a similar fashion to Sample #1 and then pyrolyzed at roughly 500 °C. Sample #3 was shredded and pyrolyzed between 500 °C and 600 °C but was only sieved after pyrolysis. All 3 approaches hold industrial relevance in an industry that has yet to settle on a standardized best practice for black mass production. Samples #1–3 were produced from industrial end-of-life battery waste streams, representing the different compositional mixtures of lithium nickel manganese cobalt oxide (NMC), organic and carbonaceous materials, and current collector materials. Sample #1, for instance, happened to be Ni-rich, while Sample #2 was Mn-rich and sample #3 was Co-rich.

**Figure 2:**
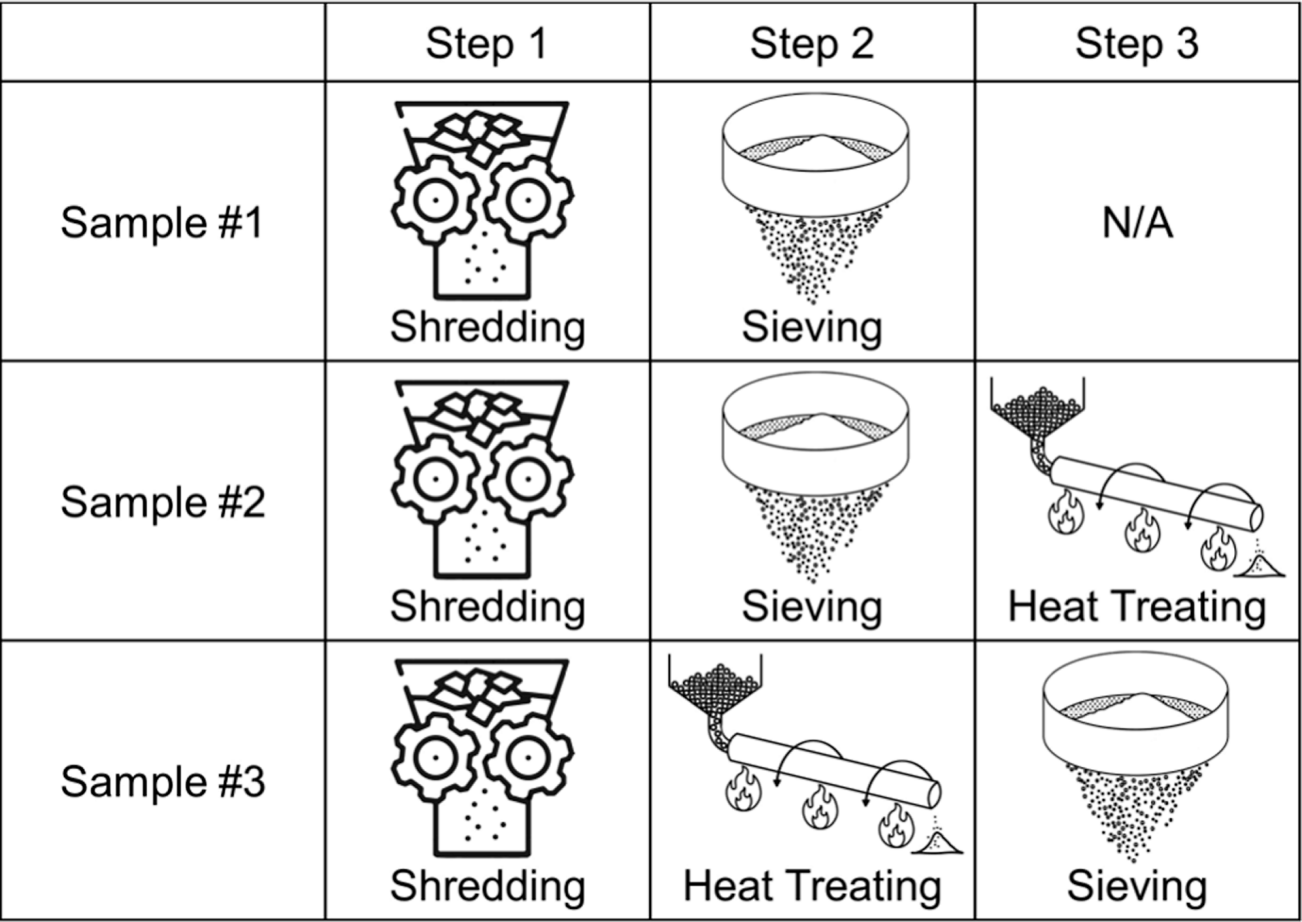
Schematic representation of the different processing steps utilized for Samples #1, #2, and #3.

#### Models of Black Mass

Synthetic proxies to industrially derived black mass were created to better understand the chemistry occurring during pyrolysis through rigorous control over reaction conditions. Synthetic proxies of black mass feedstocks were created from pairs of LIB components and subjected to a model recycling process to produce a synthetic analogue to black mass. The feedstocks contained 80 mass % pristine NMC811 cathode material (LiNi_0.8_Mn_0.1_Co_0.1_O_2_) with the remaining 20 mass % made up of one of several other LIB components, namely, graphite, LiPF_6_ electrolyte salt, and Al foil current collector. All materials were of battery grade and supplied by BASF. The model recycling process entailed cogrinding the feedstock to create a homogeneous mixture and subsequent heating in a horizontal tube furnace at 650 °C for 2 h under flowing (50 cm^3^/min) ultrahigh purity (99.999%) N_2_ (Airgas).

#### X-ray Fluorescence (XRF)

Wavelength dispersive XRF was used to investigate the elemental composition of the black mass powder. Samples were measured by using a Rigaku Primus IV Spectrometer. The instrument operated at 3.5 kW using a sealed tube Rh target X-ray source. Elemental composition results were obtained using the semiquantitative (SQX) package provided through Rigaku ZSX Guidance software. The semiquantitative package is based on the XRF FP (fundamental parameters) method using the ZSX internal sensitivity library.

#### X-ray Photoelectron Spectroscopy (XPS)

XPS was used to investigate the elemental composition of the samples. The black mass powder was spread onto double-sided tape attached to a stainless-steel sample holder. The samples were measured by using a Thermo Fisher ESCALAB Xi + XPS equipped with a monochromatic Al Kα X-ray source (1486.7 eV). Survey scans were collected at 50 eV pass energy and 150 ms of dwell time, with two scans collected and summed to improve the signal-to-noise ratio. Quantification analysis was performed using Thermo Fisher software Thermo Avantage.

#### Scanning Electron Microscopy (SEM) and Energy Dispersive X-ray Spectroscopy (EDS)

Field emission scanning electron microscopy was performed on the powder samples using a Thermo Fisher Apreo C FEG SEM instrument equipped with an EDS detector (Octane Elect Silicon drift detector, Ametek, Inc.) for chemical analysis.

#### Inductively Coupled Plasma–Optical Emission Spectroscopy (ICP–OES)

Samples were digested with a high-pressure microwave acid digestion method using the UltraClave digestion system. Three milliliter of concentrated HNO_3_ (16 mol L^–1^) was added to 100 mg of sample material before the vessel was sealed and pressurized to 40 bar and heated up to 240 °C. The solution was measured using an Agilent Technologies 5110 Synchronous Vertical Dual View Inductively Coupled Plasma–Optical Emission Spectrometer. An Sc (361.383 nm) internal standard was used. Data were normalized to exclude C and O.

#### Powder X-ray diffraction (XRD) and Neutron Diffraction

High-resolution synchrotron powder XRD (sXRD) data of all samples were collected at beamline 11-BM at the Advanced Photon Source at Argonne National Laboratory using the mail-in program.^27^ To better detect Li and to differentiate between very similar metals like Mn, Co, Ni, and Cu, the black mass samples were also studied with neutron diffraction. Samples were sent to the HB-2A high-resolution powder diffractometer housed in the High-Flux Isotope Reactor at Oak Ridge National Laboratory.^28^ Powder samples of about 6 g, held in a cylindrical vanadium container, were measured at room temperature. Measurements were performed by using λ = 0.539 A^°^ monochromatic neutron beams provided by a vertically focused Ge monochromator. The data were collected by scanning the detector array consisting of 44 ^3^He tubes to cover the total 2θ range of 7–133° in steps of 0.05°.

Both sXRD and joint neutron-sXRD Rietveld analyses were performed using TOPAS Academic.^29^ Refinements were performed against the collected data using the Rietveld method^30,31^ and structural models reported in the Inorganic Crystal Structure Database. Fundamental parameters of the beamline were modeled, including Lorentz-polarization; *la*, *lo*, and *lh* Voight coefficients; source-to-sample radius; and slit widths. The background was fitted using 8–20 term Chebyshev polynomials and peaks were fitted with the Thompson–Cox–Hastings or pseudo-Voigt profiles, depending on the phase, before refining lattice parameters. The preferred orientation of agglomerated graphite was accounted for with the inclusion of an eighth order spherical harmonics parameter. Crystal structures were visualized using VESTA version 4.0.^32^

## Results and Discussion

sXRD data were collected to identify different phases present in Samples #1–3. sXRD data of the mechanically processed but nonpyrolyzed Sample #1 are shown in Figure 3. Rietveld refinement showed this sample was composed of NMC, graphite, and a small amount of Cu (the negative current collector). The absence of Fe and Al confirmed that the mechanical processing stage was effective at separating and removing the outer battery casing from the black mass. Additionally, we observed no evidence of NMC reacting during initial mechanical processing.^33^ Vacuum drying was employed to remove volatile organic components of the electrolyte. However, LiPF_6_ salt (and its common decomposition product LiF) remained and reacted under ambient conditions, which resulted in small peaks that could not be assigned. The refined quantities of each phase in the three industrially processed samples are listed in Table S1. The final *R*_wp_ value was 11.75%.

**Figure 3:**
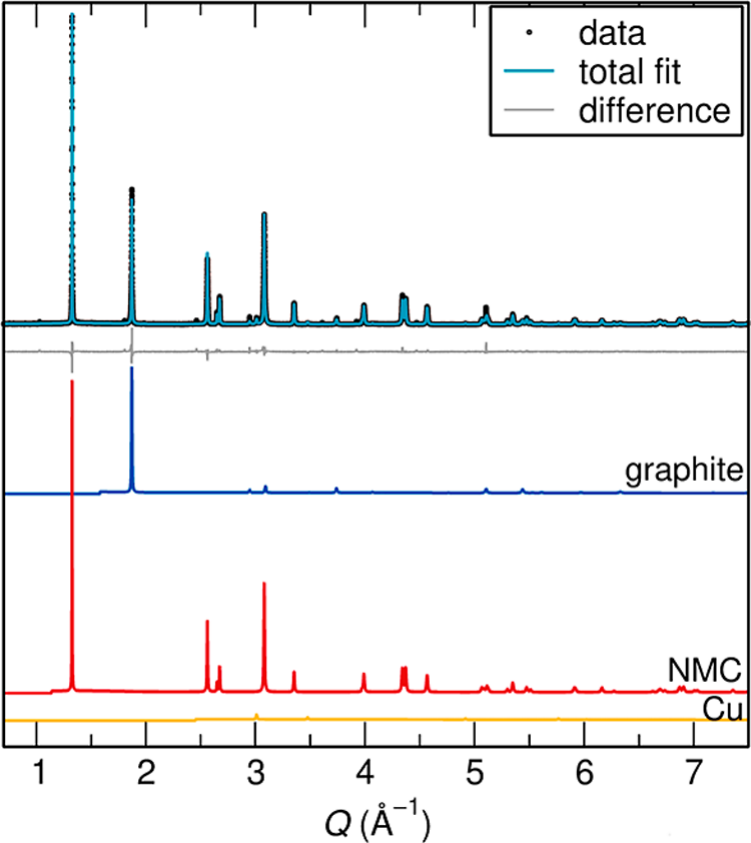
Rietveld refinement against sXRD data for Sample #1. Contributing phases are depicted, in their respective relative intensities, below the refined sXRD data.

Unlike Sample #1, Samples #2 and #3 contain phases formed at elevated temperatures during pyrolysis (Figure S1). A combination of element specific analytical techniques (XRF, ICP–OES, XPS, and EDS) was employed to quantify elemental composition. Elemental analysis helped identify possible phases present to aid in fitting scattering data collected for these samples. XRF and EDS were well suited to identifying heavier elements present in the samples, ICP–OES could probe all elements except C and O in the bulk, and XPS was capable of probing elements close to the materials’ surface. The results from Sample #2 (sieved prior to pyrolysis) are given in Table S2, and the results from Sample #3 (sieved after pyrolysis) are given in Table S3. Figure 4 compares normalized elemental ratios present in each sample.

**Figure 4:**
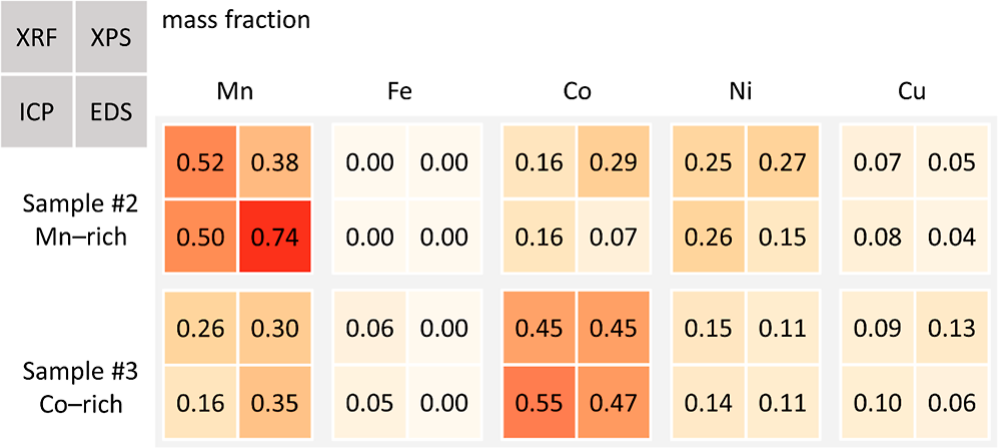
Schematic comparing the normalized elemental composition of the heavier elements (that could be measured with XRF) in Samples #2 and #3 from all four elemental analysis techniques.

Both samples contained small amounts of Cu (from the current collector) but very little Fe (from the battery casing), supporting the effective battery casing separation suggested by Figure 3. The presence of Ni, Mn, and Co was the result of NMC being the primary cathode material present in the black mass. Notably, Sample #2 was Mn-rich, whereas sample #3 was Co-rich and Ni-deficient (due to variations in NMC compositions used in commercial batteries), further highlighting the challenges posed by inhomogeneity of black mass materials collected from recycled batteries. Local SEM–EDS (Figure 5) mapping supports that these transition metals are present as their common oxides following pyrolysis. Raw EDS maps used to determine phase composition are provided in Figure S2. The presence of LiF was likely due to the decomposition of LiPF_6_.^34^

**Figure 5:**
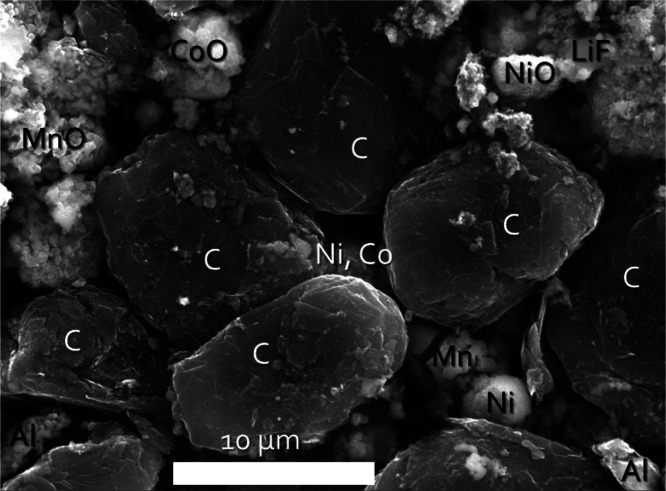
SEM micrograph coupled with EDS mapping analysis showing aggregated particles of metal oxides, metal alloys (containing no oxygen), nanoscale carbon, and LiF present in Sample #2 after pyrolysis.

Neutron diffraction and sXRD data were jointly refined for Samples #2 and #3 as shown in Figure 6. NiO, CoO, Li_3_PO_4_, HCP-Co (hexagonal close-packed), and Al were unable to be resolved in the lower-resolution neutron diffraction data (due to a lower flux of neutrons than X-rays) and thus were only refined in the sXRD data. Both samples contained significant amounts of graphite,^35^ an FCC (face-centered cubic) Ni-rich Ni–Co alloy (isostructural to FCC Ni and Co, with lattice parameters closer to Ni),^36^ metallic Cu,^37^ MnO,^38^ LiF,^39^ and Li_2_CO_3_.^40^ Additionally, in Sample #2, Li_3_PO_4_ ^41^ and a solid solution of CoO^42^ and NiO^38^ were also present (indicated by refined lattice parameters between those of pure CoO and NiO). The final *R*_wp_ values for Samples #2 and #3 were 13.23% and 16.81% respectively.

**Figure 6:**
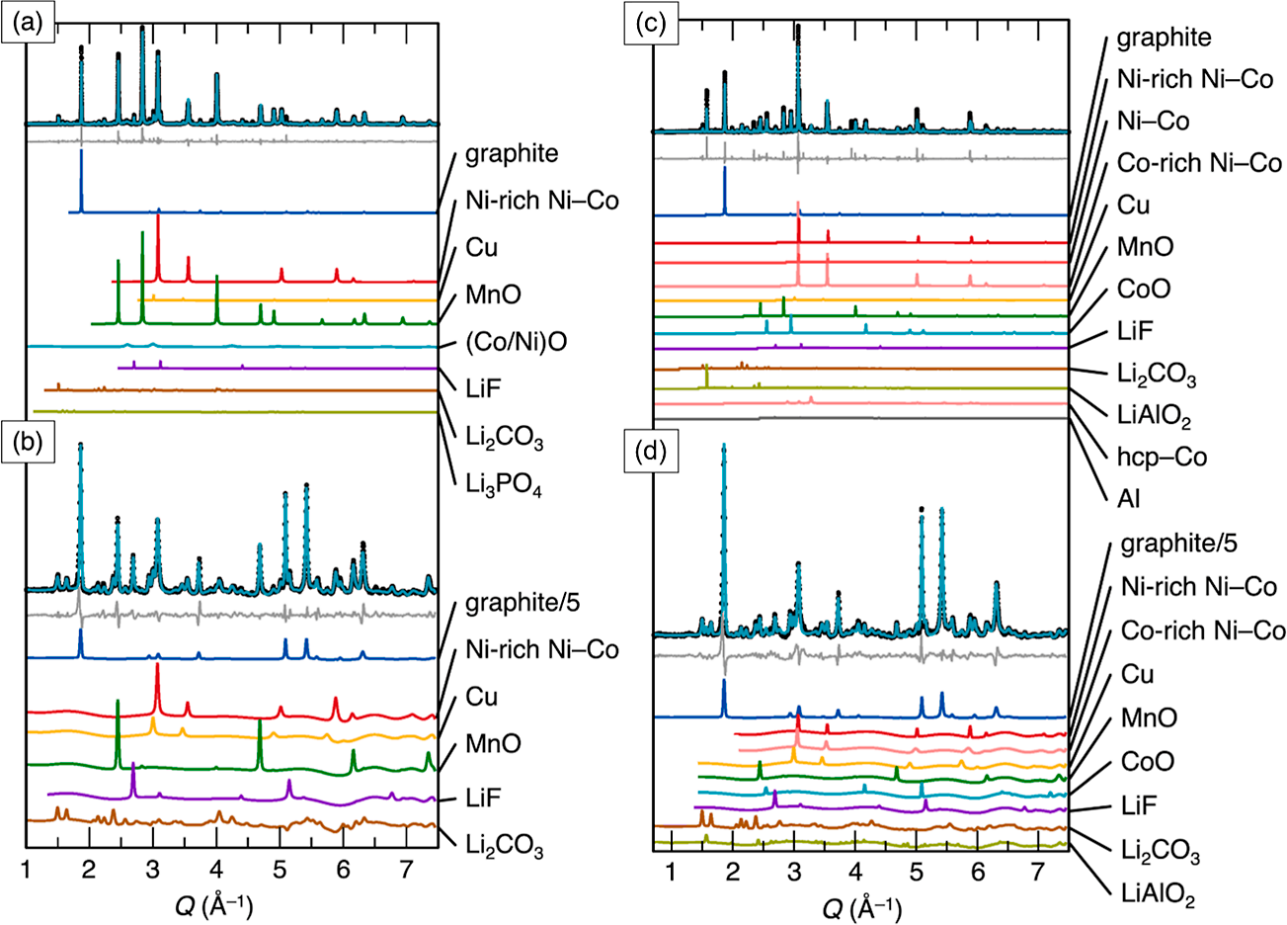
Joint neutron and sXRD Rietveld refinements for Samples #2 and #3 with individual contributing phases plotted below. In all, the gray line is the difference between the fit and the raw data. (a) Refinement against the sXRD data for Sample #2. (b) Refinement against the neutron diffraction data for Sample #2. (c) Refinement against the sXRD data for Sample #3. (d) Refinement against the neutron diffraction data for Sample #3. Note that the graphite intensity in the neutron fits was diminished by a factor of 5 for ease of plotting.

There are differences in the phases present in Sample #2 and Sample #3. Unlike Sample #2, Li_3_PO_4_ was not present in Sample #3. Instead, LiAlO_2_ ^43^ was observed in Sample #3, in agreement with elemental analysis (Tables S2 and S3) which reports the presence of more Al in Sample #3 than Sample #2. Several small peaks also indicated the presence of Al metal, a common cathode current collector material.^44^

Unlike Sample #2, in Sample #3 the FCC Ni–Co alloy was present as three distinct phases (three peaks are present in Figure 7a), suggesting three compositionally distinct alloys. However, distinct compositions would not be expected as alloying of Ni and Co is thermodynamically favorable under pyrolysis conditions.^45^ Instead, alloying may have been prevented from reaching equilibrium by kinetic limitations during pyrolysis.

**Figure 7:**
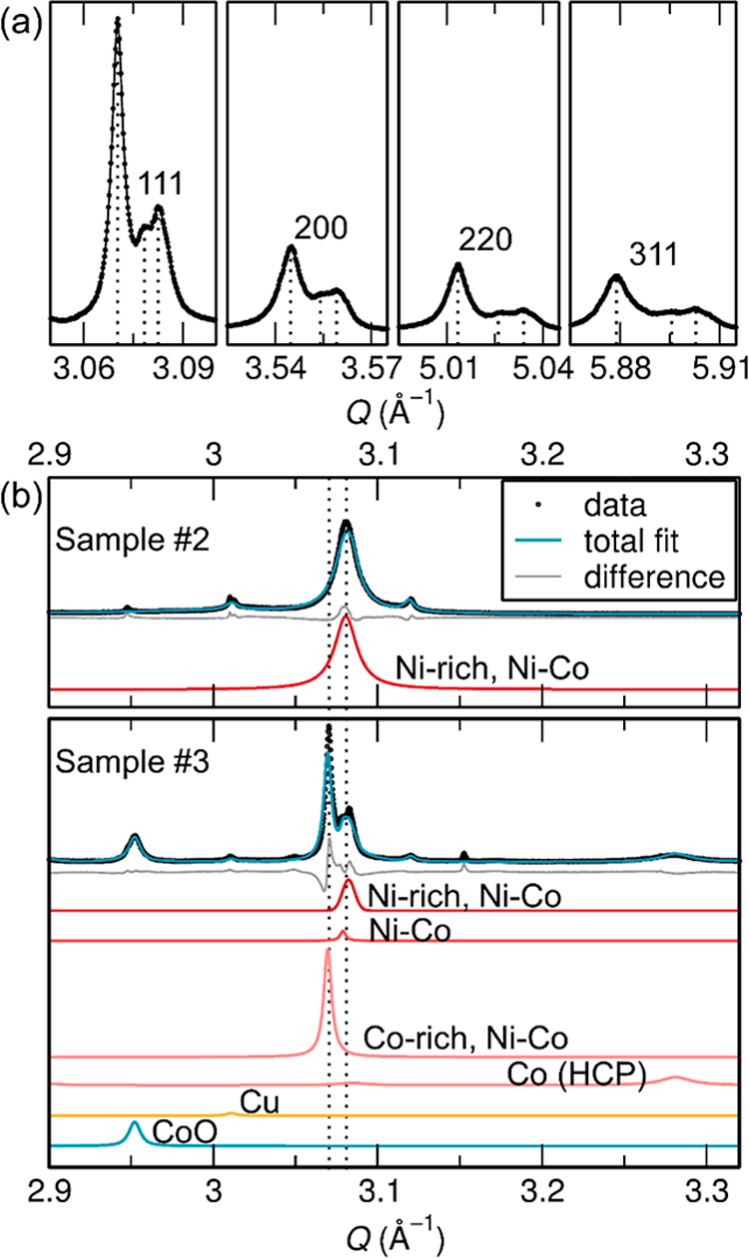
(a) The first four sets of peaks belonging to FCC Ni and Co in the sXRD data of Sample #3. Note that 3 separate alloys appear to be present as three separate sets of peaks. (b) Detailed view of the Rietveld refinements against the sXRD data from both Samples #2 and #3 to compare the angular position of their Ni–Co 111 peaks. Peak position is a result of the lattice parameter which can be used to calculate the relative amounts of Ni and Co in the alloy.

The specific Ni/Co ratios within these alloys were hard to resolve by diffraction alone as metallic FCC Ni and Co^46^ were isostructural. Ni–Co alloys follow Vegard’s Law and their approximate compositions could be calculated by extracting their lattice parameters and comparing them to the end members Ni and Co.^47^ FCC Co and FCC Co-rich Ni–Co alloys were metastable at room temperature and converted to the HCP structure spontaneously or under the application of mechanical stress.^48^ This is notable as Sample #3 was sieved and further mechanically processed after pyrolysis and was the only sample to contain HCP Co.^49^ The HCP Ni–Co alloy can only contain up to 8 atomic % Ni, which may have formed preferentially in the Ni-deficient sample #3, potentially leading to decomposition and the complex combination of phases observed.^50^

Mass % of different phases (Table S1) and their constituent elements (Table 1) was calculated from the relative intensities of peaks in sXRD and neutron diffraction data. While the quantification of phase fractions is limited by data quality and model accuracy, the calculated percentages are supported by elemental analysis in Tables S2 and S3. Using pricing information from the Shanghai Metals Market^51^ and the elemental quantification from the sXRD refinement, the economic value of each industrial black mass sample was calculated. Assuming complete recovery of Al, Co, Cu, graphite, Li, Mn, and Ni (and excluding O, F, and P which are often not recovered), the value in USD per 100 kg of each of Samples #1, #2, and #3 were $863, $1044, and $1157, respectively. The black mass value was predominantly derived from the valuable Co and Li contents, although Ni and the large fraction of graphite also contributed meaningfully. This value distribution presents a significant challenge to industrial recyclers as the cobalt content of batteries drops with the adoption of high-Ni NMC, LFP, and LMO batteries in large format applications, such as electric vehicles and stationary storage. An additional value could be derived from electrolyte, conductive salts, and binders if the recycling process was modified to incorporate their recovery. Although complete recovery is unfeasible, academic studies routinely report metal leaching recovery efficiencies of over 95%.^15^

**Table 1:** Elemental Composition (Mass %) Extracted from the Phase Percentages Approximated from the sXRD and Neutron Diffraction Joint Refinements^a^

element	sample #2 sXRD	sample #2 neutron	sample #3 sXRD	sample #3 neutron
O	28.7 ± 0.42	7.3 ± 0.21	31.5 ± 0.24	10.9 ± 0.65
Co	13.4 ± 0.23	38.7* ± 0.52	34.1 ± 0.20	36.3* ± 2.97
Ni	13.4 ± 0.23	38.7* ± 0.52	7.0 ± 0.20	33.5* ± 2.97
Mn	30.1 ± 0.25	6.5 ± 0.13	7.2 ± 0.06	4.6 ± 0.31
Cu	0.6 ± 0.02	4.2 ± 0.27	0.8 ± 0.01	7.2 ± 0.60
Al			5.8 ± 0.07	1.5 ± 0.12
Li	7.4 ± 0.13	2.4 ± 0.06	9.1 ± 0.07	3.4 ± 0.20
F	5.7 ± 0.09	2.3 ± 0.05	4.5 ± 0.07	2.6 ± 0.17
P	0.7 ± 0.04			

a Numbers are normalized to exclude C because the graphite displayed severe preferred orientation, confounding quantification efforts. *Estimation of Ni and Co metal mass from the neutron diffraction fits was divided evenly between the two because Ni and Co FCC peaks could not be well resolved in the neutron diffraction data. Sample #3 has more mass assigned to Co due to the presence of CoO.

As indicated above, a key challenge with processing black mass is the accurate identification of the complex mixture of minor phases present after pyrolysis. Given the inhomogeneous nature of mixed waste streams in the recycling process, deconvolution of the complex diffraction patterns (Figure 6) is difficult. To further our understanding of the chemistry occurring during pyrolysis of black mass and justify the phases fit in our refinement, we produced proxy black mass compositions from commercially available electrode materials. To do so, we individually reacted pristine NMC811 cathode material with graphite, LiPF_6_, and Al foils by heating at 650 °C in a tube furnace under a N_2_ flow to mimic industrial pyrolysis conditions. The resulting synthetic black mass proxies support the phases identified by Rietveld refinement, as shown in Figure 6.

Much of the chemistry observed in industrial black mass can be explained by the carbothermal reduction reaction occurring when a simple mixture of NMC and graphite is pyrolyzed.^52^ Figure 8 shows the XRD pattern of such a proxy made from the pyrolysis of 80 mass % NMC and 20 mass % graphite. The carbothermal reduction reaction in this system exhibits results in the decomposition of NMC into reduced oxides, metals, and Li_2_CO_3_, according to eq 1.

1 $${LiNi}_{x}{Mn}_{y}{Co}_{z}O_{2}+C\;\left(graphite\right)\to {Li}_{2}{CO}_{3}+NiO/CoO+Ni/Co+MnO$$

**Figure 8:**
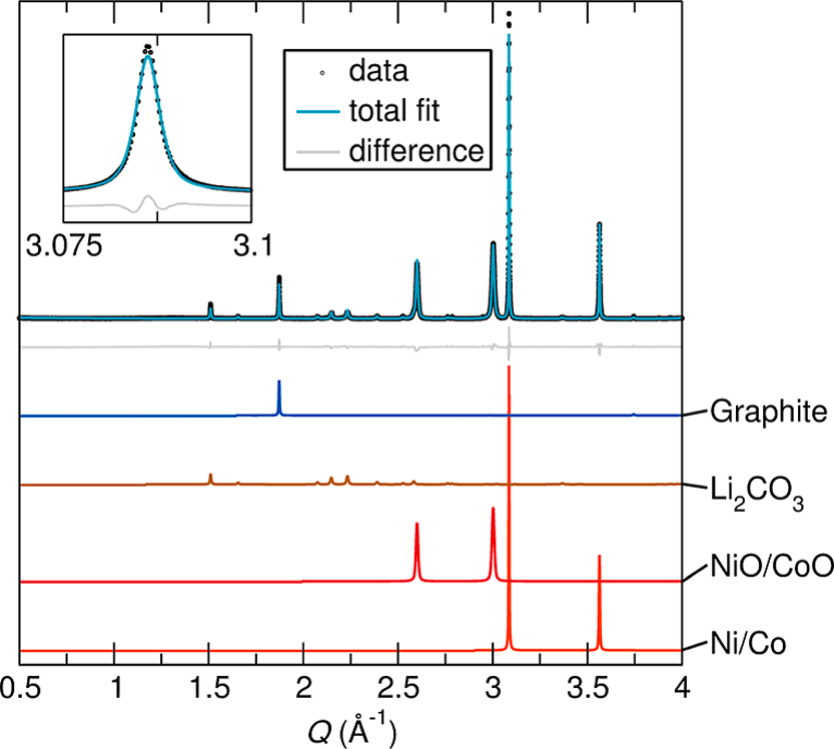
Rietveld refinement against sXRD data for a synthetic proxy of black mass made from the pyrolysis of NMC and graphite. The system was reacted in a tube furnace at 650 °C for 2 h under flowing N_2_ gas. Inset displays the first Ni–Co peak, appearing as an alloy of the two metals rather than separate phases. The phase is determined to be Ni-rich as its refined lattice parameters are between those of pure Ni and Co but closer to those of Ni.

This reaction confirms the alloying behavior of Ni and Co metals (forming a similar Ni-rich Ni–Co alloy) as well as the formation of a solid solution of their oxides. Figure 8 shows that only a single Ni–Co alloy is formed, supporting the thermodynamic favorability of alloy formation and further suggesting that more complex kinetic limitations might have led to incomplete alloying in Sample #3 (Figure 7). Further, decomposition of NMC would have produced the reactive Li_2_O species which, upon contact with CO_2_ produced from the oxidation of graphite, formed Li_2_CO_3_.^33^ MnO was not observed due to the small quantity of Mn present in the high-Ni NMC. The final *R*_wp_ value was 11.22%.

The safe processing and removal of LiPF_6_ is a clear target for the LIB recycling community due to the hazards of toxic HF and phosphates, produced upon LiPF_6_ decomposition.^34^ Figure 9 suggests that during pyrolysis of 80 mass % NMC and 20 mass % LiPF_6_ electrolyte salt, evolved HF and phosphorus oxides (P_*x*_O_*y*,_ where *x* = 1–4 and *y* = 1–9) react with Li_2_O to form LiF and Li_3_PO_4_, respectively, as trace products observed by XRD. This proxy system provided supporting evidence that these phases were present after pyrolysis of industrial black mass. LiF and Li_3_PO_4_ have limited commercial value and bring increased complexity to Li recovery. The high solubility of LiF meant a fluorine fixing agent such as Ca­(OH)_2_ was required for separation, adding further complexity due to the presence of Ca^2+^ ions that had to be subsequently removed.^53^ Li_3_PO_4_ was less soluble and was often removed by organic solvent extraction.^54^ In both cases, cost and complexity increased, making LiF and Li_3_PO_4_ undesirable during commercial LIB recycling. The final *R*_wp_ value was 14.73%.

**Figure 9:**
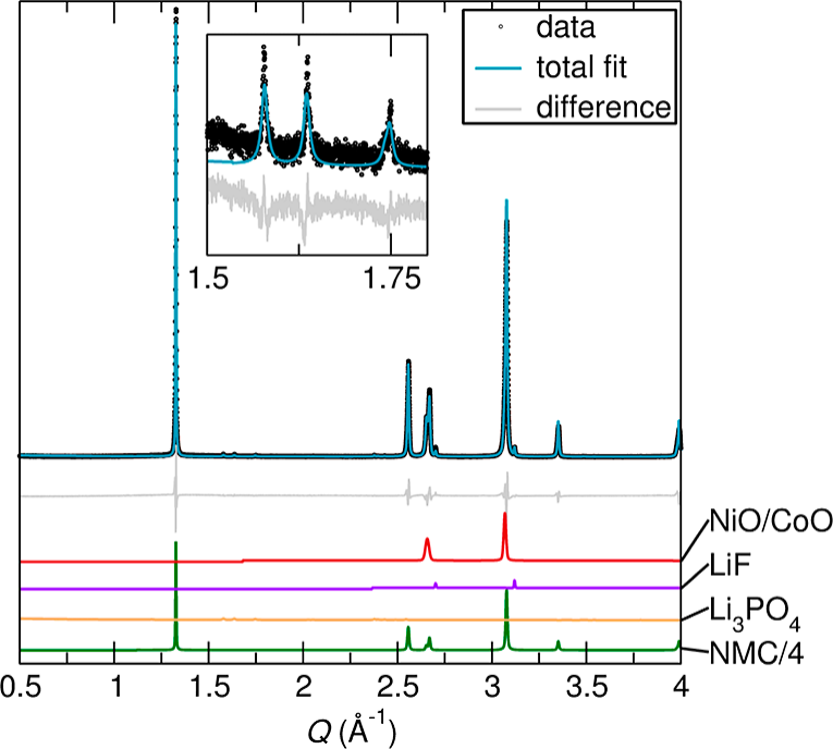
Rietveld refinement against sXRD data for a synthetic proxy of black mass made from the pyrolysis of NMC and LiPF_6_. The system was reacted in a tube furnace at 650 °C for 2 h under flowing N_2_ gas. Inset zooms in on the low intensity peaks from Li_3_PO_4_. Note that the NMC intensity was diminished by a factor of 4 for ease of plotting.

Figure 10 shows the sXRD of a final proxy system, this time comprising pyrolysis products of NMC and Al foil fragments. Here, Al acted as a reducing agent responsible for the decomposition of NMC into NiO, CoO, and Li_2_O. Because there was no carbon source, Li_2_CO_3_ could not form during pyrolysis, which allowed Li_2_O to react with the other components in the mixture to form LiAlO_2_, LiAl_2_(OH)_7_·2H_2_O,^55^ and Li_0.22_Ni_0.78_O.^56^ Li_2_CO_3_ was only formed after exposure to atmospheric CO_2_.^52^ The absence of LiAl_2_(OH)_7_·2H_2_O and Li_0.22_Ni_0.78_O in the industrial black mass samples suggested that Li_2_CO_3_ was the most stable form of lithium in carbon-containing black mass samples. LiAlO_2_ was fit to Sample #3 but not Sample #2 due to Sample #2 having most of its Al removed via sieving before pyrolysis. Similar to LiF and Li_3_PO_4_, LiAlO_2_ was not a valuable product and its recovery via separate base leaching steps increased cost and complexity.^57,58^ The final *R*_wp_ value was 9.49%.

**Figure 10:**
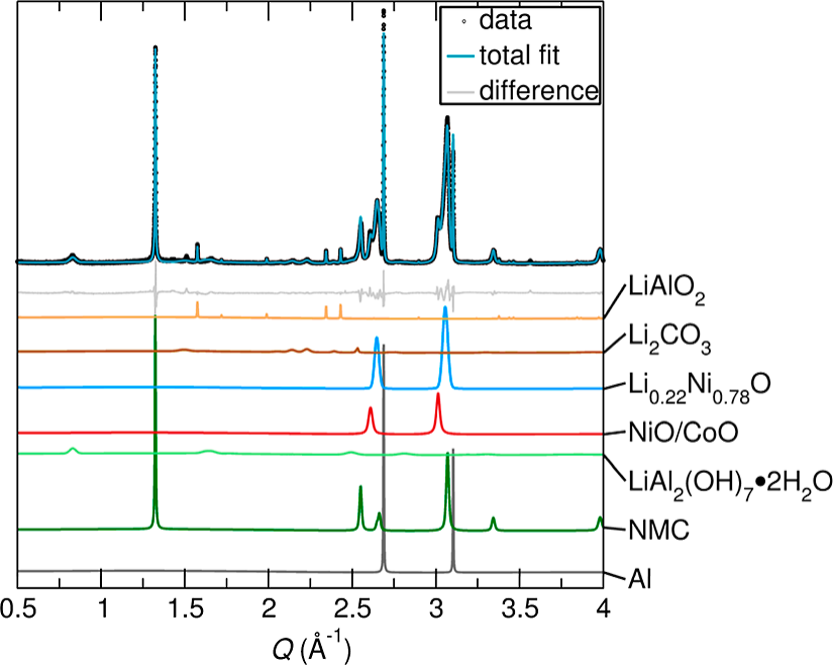
Rietveld refinement against sXRD data for a synthetic black mass proxy made from the pyrolysis of NMC and Al. The system was reacted in a tube furnace at 650 °C for 2 h under flowing N_2_ gas.

## Conclusions

A key consideration for improving recyclability of Li-ion batteries is understanding how the pyrolysis conditions affect the formation of different inorganic phases and how they can each be individually recovered. Industrial-scale waste streams produce considerable amounts of LiF, Li_3_PO_4_, LiAlO_2_, and Li_2_CO_3_. Of these, only Li_2_CO_3_ is a useful precursor for making new cathode materials.

Here, we have assessed industrially sourced black mass samples produced by different processing orders and analyzed their component phases using a suite of techniques for the determination of composition and structure. Sample #1 confirmed that industrial black mass was largely composed of graphitic carbon and NMC and that sieving was effective at removing almost all metallic casing and current collector materials. Samples #2 (sieved prior to pyrolysis) and #3 (sieved after pyrolysis) confirmed pyrolysis during industrial processing led to the decomposition of NMC via carbothermal reduction, primarily producing reduced oxides and metal alloys.^59^ The different processing strategies of these two samples represented two distinct approaches to LIB recycling. Pyrolyzing before sieving maximized the recovery of active materials by decomposing the PVDF binder and liberating active materials from the current collectors. However, we showed that this approach promoted the formation of LiAlO_2_, which was known to resist acid leaching and required complex additional steps to recover Li.^57,58,60,61^ Pyrolysis after sieving prevented the formation of LiAlO_2_ by removing Al foil current collectors before they could react with Li_2_O. This came at the expense of reduced active material recovery, as some active material particles were invariably attached to the current collectors and sieved out.

Additionally, we showed that the employment of simple model systems as proxies to industrial systems allowed us to better identify minor phases likely formed during thermal treatment of commercial recycling streams. Furthermore, this approach provided insights into possible phases that lithium can take in the absence of carbon. Producing synthetic black mass proxies to examine minor product phases presents a possible avenue for further Li recovery applicable to processes beyond the laboratory scale. We are currently expanding this approach to investigate the recycling implications of emerging cathode materials in future work.

## Supplementary Material



## Notes

Tabular values for phases refined in Samples 1–3, XRD plots of Samples 1–3, tabular values for elemental analysis (XRF, ICP, XPS, and EDS) on Samples 2 and 3, and EDS maps of Sample 2 (PDF)
