# Supplementary material for: Pyrolyzed “Black Mass” Feedstocks and Their Synthetic Proxies Relevant to Li-Ion Battery Recycling
Source: ACS Omega. 2025 Jun 10;10(24):25546–54. doi: 10.1021/acsomega.5c00995 (PMC12198988; doi:10.1021/acsomega.5c00995)

Supporting Information for
Pyrolyzed "Black Mass" Feedstocks and their Synthetic Proxies Relevant to Li–Ion Battery Recycling

Alexander J. Bologna,[§,‡,¶] Rebecca C. Vincent,[†,‡,¶] Anna Kallistova,[‡]
Justin A. Mayer,[†,‡] Matthew A. Wright,[†,‡] Clarina R. Dela Cruz,[||] Rui Zhang,[⊥]
Fabian Seeler,[#] Kerstin Schierle-Arndt,[#] and Ram Seshadri [*,†,‡,§]

§Department of Chemistry & Biochemistry, University of California
Santa Barbara, California 93106, United States
‡Materials Research Laboratory, University of California
Santa Barbara, California 93106, United States
¶Contributed equally to this work
†Materials Department, University of California
Santa Barbara, California 93106, United States
|| Neutron Scattering Division, Oak Ridge National Laboratory 1 Bethel Valley Rd., Oak Ridge, Tennessee 37831, United States
⊥California Research Alliance (CARA), BASF Corporation
Berkeley, California 94720, United States
#BASF SE, 67056 Ludwigshafen, Germany

Table S1: Refined quantities (mass-%) and corresponding errors for each phase fit in the synchrotron x-ray and neutron data of industrially processed Samples #1, #2, and #3.

Sample #1 sXRD	
Phase	Quantity
Graphite	45.9 ± 0.32
NMC	53.3 ± 0.32
Cu	0.9 ± 0.02

Sample #2 sXRD		Sample #2 neutron	
Phase	Quantity	Phase	Quantity
Graphite	20.3 ± 0.39	Graphite	3.5 ± 0.08
Ni-rich Ni-Co	15.9 ± 0.18	Ni-rich Ni-Co	73.9 ± 0.52
Cu	0.5 ± 0.02	Cu	3.8 ± 0.27
MnO	29.6 ± 0.32	MnO	8.3 ± 0.16
(Co/Ni)O	5.8 ± 0.14	LiF	2.4 ± 0.07
LiF	5.6 ± 0.12	Li_2CO_3	7.9 ± 0.31
Li_2CO_3	20.1 ± 0.76	-	-
Li_3PO_4	2.3 ± 0.13	-	-

Sample #3 sXRD		Sample #3 neutron	
Phase	Quantity	Phase	Quantity
Graphite	33.3 ± 0.55	Graphite	15.1 ± 1.06
Ni-rich Ni-Co	3.8 ± 0.08	Ni-rich Ni-Co	1.8 ± 0.32
Ni-Co	0.9 ± 0.06	Co-rich Ni-Co	54.4 ± 3.19
Co-rich Ni-Co	13.8 ± 0.14	Cu	5.7 ± 0.60
Cu	0.3 ± 0.01	MnO	5.0 ± 0.40
MnO	6.3 ± 0.08	CoO	3.1 ± 0.44
CoO	6.9 ± 0.08	LiF	2.9 ± 0.24
LiF	4.3 ± 0.10	Li_2CO_3	8.8 ± 0.66
Li_2CO_3	20.6 ± 0.35	$LiAlO_2$	3.3 ± 0.30
$LiAlO_2$	7.7 ± 0.15	-	-
hcp-Co	1.6 ± 0.02	-	-
Al	0.5 ± 0.04	-	-

Figure S1: Raw synchrotron XRD data for Samples #1, #2, and #3. Even prior to Rietveld refinement, it is evident that Samples #2 and #3 contain phases that are not present in the unheated Sample #1.

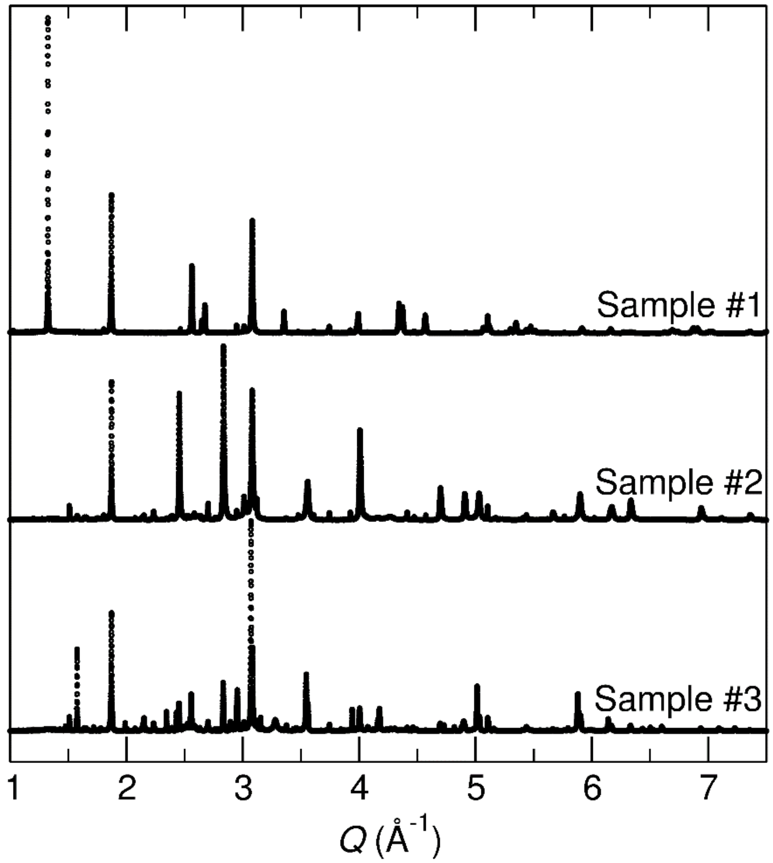

Table S2: Elemental analysis (mass-%) of Sample #2 via four different techniques: X-ray fluorescence (XRF), inductively coupled plasma optical emission spectroscopy (ICP), X-ray photoelectron spectroscopy (XPS), and energy dispersive X-ray spectroscopy (EDS). Standard errors are given for XRF and ICP, but not for XPS and EDS due to the surface sensitive nature of the techniques and the non-homogeneity of the sample.

Element	XRF	ICP	XPS	EDS
C	32.0(4)	–	66.3	58.8
O	30(1)	–	14.7	20.0
Co	4.3(2)	11.83(1)	1.3	1.1
Ni	6.8(3)	19.40(1)	1.2	2.2
Mn	14.3(6)	37.83(3)	1.7	11.2
Cu	1.8(1)	5.82(4)	0.2	0.6
Al	4.46(9)	6.79(2)	4.1	2.6
Fe	0.07(1)	0.14(2)	–	–
Li	–	7.57(1)	5.3	–
Na	–	–	0.1	–
F	5.2(4)	8.5(1)	2.8	3.0
P	0.84(3)	1.63(6)	2.2	0.3
Si	–	0.17(9)	–	–
Mg	0.09(1)	0.29(3)	–	–

Table S3: Elemental analysis (mass-%) of Sample #3 via four different techniques: X-ray fluorescence (XRF), inductively coupled plasma optical emission spectroscopy (ICP), X-ray photoelectron spectroscopy (XPS), and energy dispersive X-ray spectroscopy (EDS). Standard errors are given for XRF and ICP, but not for XPS and EDS due to the surface sensitive nature of the techniques and the non-homogeneity of the sample.

Element	XRF	ICP	XPS	EDS
C	45.1(3)	–	57.5	75.0
O	31.1(9)	–	20.1	14.2
Co	7.1(4)	38.91(2)	2.1	2.4
Ni	2.4(1)	9.58(2)	0.5	0.6
Mn	4.0(2)	11.57(9)	1.4	1.8
Cu	1.37(4)	6.79(3)	0.6	0.3
Al	3.4(1)	11.18(1)	7.8	2.3
Fe	0.88(4)	3.79(6)	–	–
Li	–	7.18(8)	5.1	–
Na	0.06(3)	–	0.4	–
F	2.8(1)	5.2(2)	2.8	3.0
P	0.62(3)	0.72(1)	1.7	0.4
Si	0.66(2)	4.39(3)	–	–
Mg	0.16(1)	0.38(2)	–	–
Ca	0.13(1)	0.32(2)	–	–
S	0.089(4)	–	–	–

Figure S2: Raw EDS mapping of elements used to ascribe phase compositions to Figure 5.

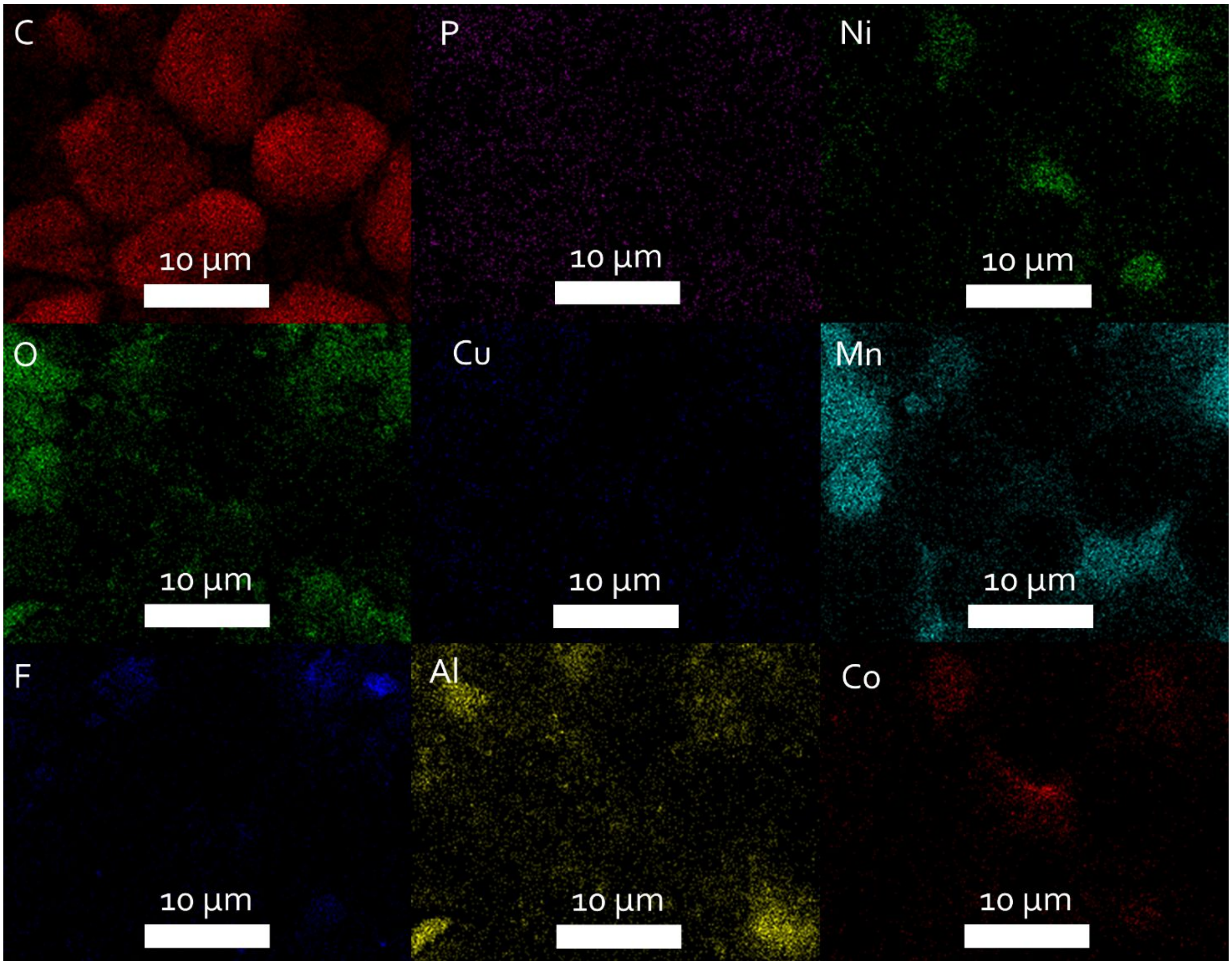

Supplement: Supplementary file 1 [file ao5c00995_si_001.pdf]
